# An evidence-based nutrition education programme for orphans and vulnerable children: protocol on the development of nutrition education intervention for orphans in Soweto, South Africa using mixed methods research

**DOI:** 10.1186/s12889-019-6596-5

**Published:** 2019-03-14

**Authors:** Temitope Kayode Bello, Jace Pillay

**Affiliations:** South African Research Chair: Education and Care in Childhood, Department of Educational Psychology, Faculty of Education University of Johannesburg, Soweto Campus, B Ring 415 / RS, Soweto, 204 South Africa

**Keywords:** Nutrition education, Malnutrition, Orphan, Vulnerable children, HIV, Quality of life, Academic performance

## Abstract

**Background:**

Focus on interventions for orphans and vulnerable children (OVC) in South Africa on education, quality of life (QoL) and nutrition-related matters have been reported diminutive. The risk of dropping out of school for an OVC with poor QoL and without varied food intake is very high. The problem with poor; QoL, nutritional care and academic performance (AP) of the OVC is that it sets the foundation for their adults’ life. The purpose of this longitudinal study is to develop, implement and to test the efficacy of an evidence-based nutrition education programme (NEP) for OVC that will integrate their families/caregivers, schools and communities.

**Methods:**

A longitudinal study, and a mixed-methods approach steered by action research will be used. This study will be in three phases. Phase 1 will be the needs assessment; Phase 2 will be the development of nutritional education materials, and Phase 3 is the intervention. QoL, dietary intakes, body composition, and anthropometric status, physical activities, and AP of 520 OVC in Soweto will be assessed using standard techniques. Nutrition knowledge, attitude and practices (KAP) of the caregivers will be assessed using previously validated questionnaires. Focus group discussion (FGD) will be conducted to gain an in-depth understanding of what OVC eat and factors affecting their food intakes. Data will be collected at baseline, week 12 and week 24. Generalised Least Squares (GLS) regression model will be used to test the study hypotheses. Atlas-ti and Thematic Framework Analysis (TFA) will be used for qualitative data analysis.

**Discussion:**

This study will provide detailed information on the QoL, food intakes concerning academic performance and general well-being of OVC in an Africa setting. The participatory mixed methods nature of the study will provide valuable insights into the drivers and challenges to QoL, AP, and nutritional status of this group. This approach will assist the policymakers’ and other stakeholders in decision making regarding the general well-being of the OVC.

**Trial registration:**

ISRCTN12835783. Date registered 14.01.2019.

## Background

The World Health Organization in 2016 reported that 15,000 deaths occurred among children per day translating into 5.6 million annually [1, 2]. More than 50% of the deaths are preventable using appropriate and evidenced-based intervention [3, 4]. Numerous scientific reports have described malnutrition as the key underlying and sensitive risks factors of vulnerability to early grave among the children [2].

Global provision of appropriate health care for orphans and vulnerable (OVC) children is critical for maximising their health well-being, potential, and quality of life (QoL) [4, 5].

Although there is considerable progress between 1990 and 2017 in reducing child mortality rates from 5.1 million to 2.6 million, it is unfortunate that the rates of progress have been reported lower than the rates of the negative impacts that children’s mortality rates have on the economic growths of the 52 nations of the world.

Nutrition-related factors contributed to 45% of deaths among the children [2].

Various interventions have been proposed and implemented in different parts of the world for OVC [6, 7]. The need to improve the nature of varied food intake among OVC in Africa has well been documented [8]. Unfortunately, the use of an evidence-based nutrition education programme (ENEP) as a component of reducing malnutrition and child mortality has not been explored fully in most of the African countries such as South Africa.

There is evidence that nutritional knowledge of caregivers/families is positively associated with the nutritional status of the children under their custody [9]. This may be the reason why nutrition-related factors were reported to contribute to 45% of deaths among the children [2].

Therefore, exploration of the risk factors that may be responsible for children to be vulnerable may be appropriate in designing more effective nutrition education intervention against malnutrition. An in depth understanding of the factors that can prevent OVC from attaining their potentials in life, such as QoL, dietary intakes, physical activities and anthropometric status concerning academic performance will be useful in developing and implementing nutrition education programme (NEP) for OVC.

Several factors can result in a child being characterised as vulnerable such as parent’s financial constraints, consistent domestic conflicts, malnutrition, unwanted by parents or caregiver, orphaned by the death of either or both parents as a result of natural deaths or diseases such as HIV [10]. Part of the worst-case scenario that can make a child vulnerable is when a child contact disease such as HIV after birth while the parents are HIV-negative. South Africa has 3.7 million orphans, and more than 50% of them were made orphans as a result of HIV/AIDS [1]. According to UNICEF reports, as at the end of 2016, South Africa has the second highest number of orphans and vulnerable children (OVC) in the world [10].

In South Africa where the mortality rate of OVC is high, a NEP to assist caregivers/ families to make the right food choices has not been exploited fully. Although there is an existing national policy framework on care and support for OVC in South Africa, there seems to be limited data on nutrition-related information and its application either in counselling or educating the caregivers/families of OVC in the framework [11]. The kind of foods given to OVC depends on the nutrition knowledge of the caregiver/family which can influence the academic achievement of the child.

Academic attainment is described as an outcome of academic performance which is associated with long-term educational feat [12, 13]. It has a vital role in determining health status and life opportunities of OVC by influencing their employment prospects, socio-economic power, QoL and other psychosocial well-being. Children exposed to insufficient or unvaried diets are more likely to encounter academic challenges. Some of the existing literature that examined the relationship between quality of food intake and academic performance reported links between quality of food, poor academic performance, learning capability, physical activities and QoL in both children and adults [12, 14, 15]. These findings suggest that what students eat may affect their academic performance. No review has synthesised the literature investigating the effects of quality of food intakes on the academic performance of OVC in South Africa. Consequently, this study objectives are to (1) document association between OVC’s QoL, individual dietary diversity scores (IDDS), physical activities, body composition and anthropometric status, academic performance and nutrition knowledge, attitude and practices of the caregivers (2) develop NEP based on the outcome of the first objective (3) implement the NEP and examine the impacts on the measurable incomes. The aim of the current study now becomes the development, implementation and impact evaluation of an ENEP for orphans and vulnerable children that will integrate their families/caregivers, schools and communities. It is noteworthy, to know that, the current research protocol premised from the broader study under the incumbent of South African Research Chair in Education and Care in Childhood awarded by the South African National Research Foundation. The incumbent Chair’s research ultimately focuses on developing intervention programmes to reduce the factors of vulnerability and enhance protective factors among OVC with the support of their families/caregivers, schools and communities.

## Methods

### Design

This longitudinal study will be in three phases. The study phases will be conducted consecutively because the phases will be mutually dependent. Phase one will be exploratory descriptive using a mixed method research approach in the qualitative and quantitative research domains. The first phase will provide information that will guide Phase 2 and Phase 3. Phase 2 will be the development of nutrition education materials based on the outcome of phase one. The third phase is an intervention in a quasi-experimental domain.

**Fig. 1:**
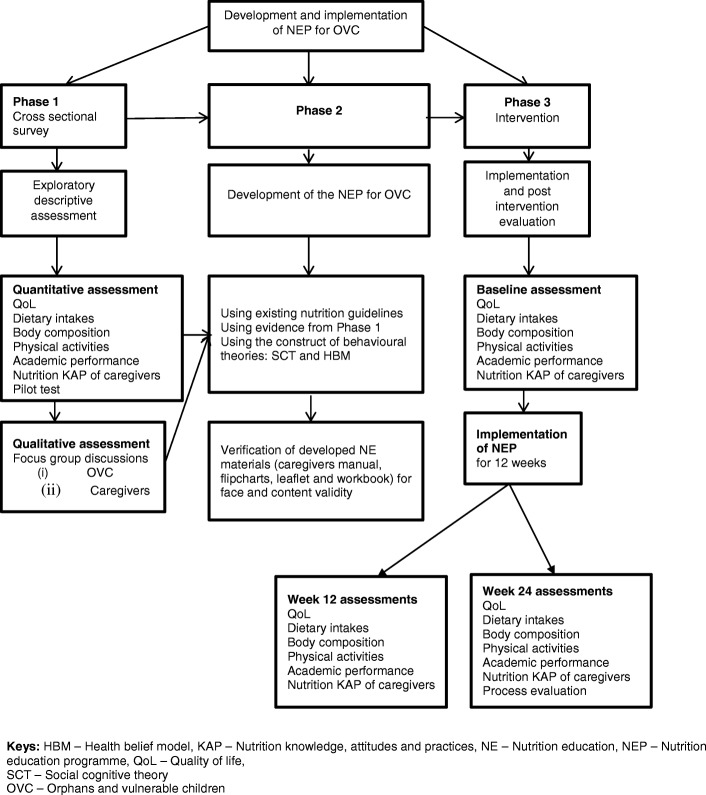
Overview of the study protocol

**Fig. 2:**
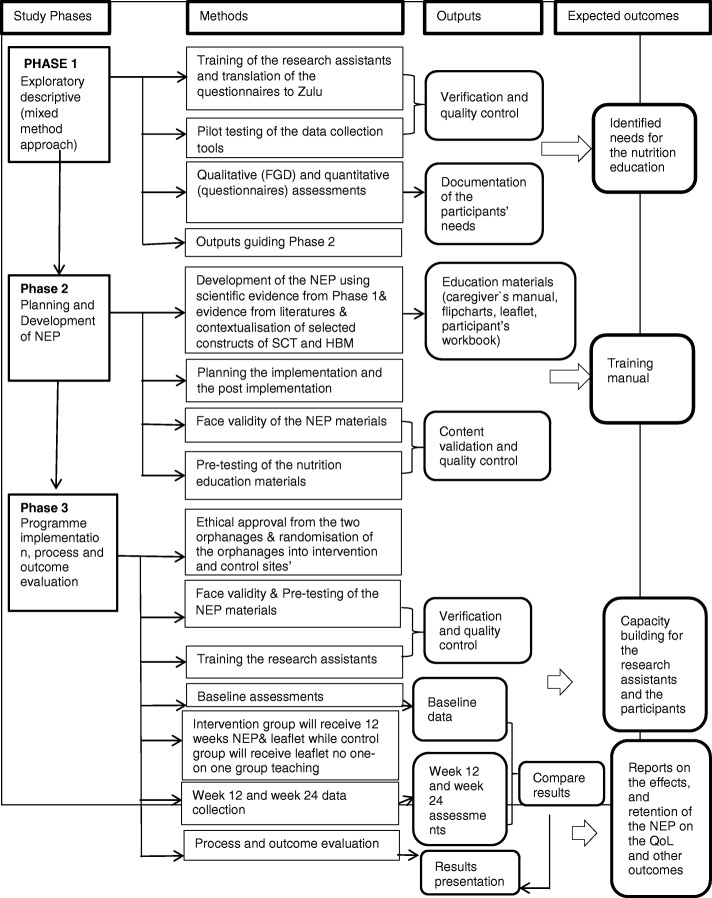
Conceptual frameworks for developing and implementing a nutrition education programe for OVC

The overview of the study is illustrated in Figs. 1 and 2.

### Settings and sampling

The study sites will be in selected schools in Soweto as stated in the broader study of the research chair in the application submitted to the Department of Science and Technology, National Research Foundation and the Faculty of Education Research Ethics Committee of the University of Johannesburg. Convenience sampling will be used to recruit the participants for Phase 1 and 3 of the study. Convenience sampling means to recruit participants who are willing and ready to give their oral and written consents during the data collection.

### Sample size

The sample size in this study was estimated based on the expected number of consenting participants in the two groups (control and the intervention group). This study desired 80% power (Z_β_ = 0.84) to estimate sample size with confidence interval (Z_∝/2_ = 1.96) of 95% as used in previous studies [16]. The investigator desires to detect a difference (d) of 5 points on a 100 point scale of QoL constructs between pre and post intervention [16, 17]. Standard deviation (*σ*) of 10 is expected in the QoL constructs between pre and post intervention [17]. Sample size (n) of 63 OVC per group (control and the intervention) to make a total of 126 OVC using the formular below is projected [18, 19].

*n* = 2(*σ*)2(*Z*_*β*_ + *Z∝*/2)2 ÷ *d*2

In order to compensate for non-response and drop out, 100% will be added to make a sample size of 126 anticipated consenting participants per group to make a total of 252 participants [18]. For the need’s assessment (Phase 1) a total of 252 is appropriate but 520 is proposed in order to further compensate for non-response and drop out.

### Recruitment and retention of participants

Recruitment of participants will start during the start of the second term. We estimated that a total of 520 students aged (12–17 years) will be selected. Participants will be chosen from secondary schools in Soweto. The OVC will be followed up at their various orphanages or through their caregivers.

### Eligibility criteria

Informed consent of school teachers will be obtained for school participation and if a school declines, a nearby alternative school will be approached. Students in the participating schools will be invited for briefing if they meet the following criteria:

1. Are within the age range of 12–17 years
2. Lost one or both parents
3. Give both oral and written consents

### Measures

All the measures are composed of items adapted from published studies. The QoL questionnaire that will be used in this study has previously been validated with acceptable psychometrics values.

### Primary outcome

QoL will be measured using Kidscreen health-related QoL questionnaire for children and adolescents. This questionnaire has previously been validated in South Africa and shows acceptable psychometrics reliability of 0.76–0.81 [20].

### Secondary outcomes

The following data will be collected to assess secondary outcomes.

#### (*a) Dietary intake assessments*

- ***Food intakes using photovoice***

The method of data collection here will be emancipatory participatory approach using photovoice and photo-assisted focus group discussions. Photovoice is a qualitative research method that enables participants to be involved in data collection by representing their experience, perception, concepts, and voices using photographs [21, 22]. The photographs will be used to capture participants’ viewpoints in the form of storytelling. It will enable participants of thisresearch to contextualise the photographs and have shared multiple viewpoints by explaining what the photographs mean to them during assisted group discussions [22–24]. A disposable camera with limited number of films will be provided to the caregivers/families of the OVC to take pictures of foods and environment where foods are provided to the OVC [25]. Participants will be trained in the use of the disposable camera. The caregivers/families OVC will be required to take pictures of food intakes OVC for three days, one of which must be weekend (Training will be provided). Such caregivers/families of OVC will be engaged in photo-assisted focus group discussions that is, they will be expected to explain what the pictures mean to them.

It will be clearly stated to the participants that, there will be no pictures of people but pictures of foods and environment only. The pictures will be used to initiate dialogue on the food intakes and food security of the OVC. The cameras will be recycled immediately the appropriate pictures have been selected. The length of the interview is estimated to be 35 min with another 25 min for discussion on the pictures taken. The group discussions will engage participants in reflective perspective analysis on the healthiness of the foods in which the photograph is taken during the three-day period.

The needs that will be identified in the food intakes of the OVC will be incorporated into the NEP that will be developed and implemented to capacitate the caregivers/families of OVC.

The photovoice will be followed with photo-assisted focus group discussions.

Photovoice is well documented in disciplines such as anthropology, health, and sociology [26, 27]. The process has been reported to be effective in arriving at more valid and reliable evidence-based data collection [26]. Experience from verbal expression may be challenging to measure quantitatively, that does not mean that quantitative data are not reliable [28]. Therefore, photovoice will be combined with a single day 24 h recall to add richness and comprehensiveness to the method of data collection [29].

- ***Food intakes using 24-h recall***

A single day non-quantified 24-h recall of OVC will be obtained from the participants via their caregivers/families. The recall will cover all days in a week. The individual dietary diversity score (IDDS) of the OVC will be used to measure the nutritional quality of the reported intake of the OVC [30–32]. The IDDS is a simple count of food groups that an individual has consumed over the previous 24 h. When the participants’ recall is complete, the foods will be classified according to the Food and Agriculture Organization of the United Nations (FAO) food groups and scored on a scale of Yes (1) or No (0) [31]. If a food item from the group is consumed, it will score ‘1’ and if not it will score ‘0’. The nine food groups will be (i) cereals, roots and tubers (ii) dark green leafy vegetables (iii) other vitamin A rich fruits and vegetables (iv) other fruits and vegetables (v) legumes and nuts (vi) meat and fish products (vii) organ meat (viii) milk and milk products (ix) eggs. The IDDS is sensitive to assessing the change in diet before and after an intervention to detect any improvement [31].

#### (b) Body composition and Anthropometric measurements

Tanita Dc-430 Body Composition Analyser (BCA) will be used to measure participants weight and body composition. Participants will be allowed to stand on the BCA without shoes or socks. Participants’ age, gender, and height will be entered into the BCA for calculations. The equations proposed by Clasey et al., for estimating the body composition of young children will be used to calculate the percentage of OVC’s body fat [33].

Weight and height of the OVC will be measured using standard techniques. Weight will be captured when an OVC stands on the BCA. Height will be obtained by allowing the participants to stand barefooted with their feet and heels together, their buttocks and upper parts of the back touching the portable stadiometer. The researcher and the trained research assistants will ensure the participants’ shoulders are relaxed with arms at the sides. While the participants will be instructed to take and hold a deep breath as the head is expected to be in straight position. The researcher will position the headboard of the device (portable stadiometer) on the participant’s head. Participants’ Body mass index (BMI) will be calculated as the ratio between the weight (kg) and height (m^2^).

The BMI of the OVC will be used to measure the occurrence of central lipohypertrophy and peripheral lipoatrophy which are significant problems among HIV-infected population. It will also be used to determine the risk of malnutrition (overweight and underweight) among the OVC.

The methods described by the American College of Sports Medicine [34] will be used to measure the Triceps and skin folds of the OVC.

#### (c) Physical activities measurements

The OVC will be given an accelerometer to wear on the waist for seven consecutive days [35–37]. Although, the cut-off point for the moderate to vigorous physical activities for children varies in the literature [38, 39]. Smith et al., proposed moderate physical activity of 1535–3961 counts per minute and vigorous physical activity of ≥3962 counts per minute [40]. For the sake of this study, this model will be used [40]. The non-wearing period will be zero counts for ≥2 h and will be excluded from activities analysis [40–42].

#### (d) Academic performance

Academic performance will be obtained from standardised source via the participants’ school administrators or national academic records [43]. In this study, the most commonly reported assessed academic subjects (mathematics, reading, writing, life science) as reported in previous studies will be assessed [43]. The GPA of the selected subjects will be calculated with 80% as the cut-points for excellent academic performance.

#### (e) Nutrition knowledge, attitude, and practices of the caregivers

An interviewer-administered validated nutrition KAP questionnaire will be used for assessing the nutrition KAP of the caregivers of the OVC. The nutrition knowledge questionnaire has previously been used among adults in South Africa settings [44, 45]. The nutrition knowledge questionnaire has three sections. Section A consists of 60 questions with different subsections on nutrition knowledge [45] questions. Subsection A1 (17 questions) measures meal plan in which participants are required to choose an answer, while subsection A2 (7 questions) assesses food preparation with mostly true or false option questions. Subsection A3 (1 question) evaluates food purchasing knowledge of the caregivers and subsection A4 (35 questions) examines general nutrition knowledge.

Likewise the attitudes (Section B) and practices (Section C) questionnaires have been previously used among adults in India [46], as well as in a resource-limited setting of Kwazulu-Natal, South Africa [44]. Section B (8 questions) consists of questions on nutrition attitudes. All the questions have three options: Agree (1), disagree (2) and don’t know (3). Section C has three subsections: subsection C1, nutrition practice using FAO food groups. Participants will be asked questions on how often the nine food groups were eaten in the previous week. Information on the caregivers’ smoking and alcohol habits will also be obtained with questions from subsection C2 (two questions on smoking habits) and subsection C3 (2 questions on alcohol use).

### Planning and development of the nutrition education programme

The needs of the OVC will be identified in the results of Phase 1. The results also will guide the contents of the NEP in Phase 2. The aim of Phase 2 will be to develop an NEP tailored to the needs of OVC using the results from Phase 1, as well as existing guidelines and information from the literature.

The development process will be guided by the steps for designing a theory based NEP [47, 48]. The steps as described by Sahyoun et al., [48] and Contento, [47, 49, 50] will be contextualied, which include:

- Identification of needs and problems analysis.
- Stating the goals and objectives of the NEP.
- Identification of barriers to healthy behavior.
- Identification, selection, and contextualization of behavior/learning theories.
- Programme components selection and identification of the key educational goals.
- Preparation for the implementation of the NEP.
- Pre-testing of the nutrition education materials.
- Process and outcome evaluations.

### Intervention

The intervention will entail the implementation of the NEP at the orphanage homes. Selected constructs of the SCT and HBM will be incorporated to enhance learning, and positive changes in attitudes and dietary behavior to improve QoL, dietary diversity, physical activities, anthropometric status and academic performance of the OVC. The NEP will address the importance of nutrition in improving QoL and the secondary outcomes, and provide guidance on how to overcome the barriers to healthy eating to enable the caregivers of the intervention group to adopt typical behaviors [49, 51].

The intervention participants will receive nutrition education materials and the 12-week NEP. The NEP is proposed to consist of:

(i) a NE trainer’s manual
(ii) a participant’s workbook to be used at home by the intervention participants to revise the topics that will be taught. This proposal is expected to enhance participants’ confidence in performing acquired knowledge and skills [49, 51].
(iii) flipcharts for pictorial demonstration; so that participants will compile evidence through visual literacy.
(iv) a leaflet summarising the NEP for self-learning.

An attendance register will be kept for follow up and measure of attrition rates.

### Data procedures

Before the needs’ assessment data collection, a series of training sessions will be held to train the research assistant responsible for taking measurements, including QoL, dietary intake assessments, body composition, and anthropometric measurements, physical activities and nutrition KAP of the caregivers. The training will be for three weeks, to be held at the South African Research Chair: Education and Care in Childhood, Faculty of Education University of Johannesburg project office. To ensure standardisation and quality of data collection, this training will include an overview of all the measurement procedures in the protocol, and demonstration of the measurement techniques. The questionnaires will be subjected to face validity, such as clarity, simplicity, and readability of the content of the questions. This process will be conducted by the principal investigators [52, 53]. The questionnaires will be pretested following the procedure described above among at least 50 conveniently sampled OVC and ten caregivers. The reliability test will be done (using Cronbach alpha) to measure the internal consistency of the questionnaires. Anthropometric data will be collected individually using the school clinic. The dial indicator of the BCA will be checked regularly and adjusted to the appropriate zero reading position before use. A non-stretchable tape measure will be used in measuring the MUAC. To ensure reliability, measurements will be done two times for each participant [54].

### Data management and analysis

The quantitative data will be entered into EXCEL (version 15; 2016), and data will be cross-checked by re-entering the data on a separate spreadsheet for comparison. Double data entry will be done to ensure the quality of the data. Data will be analyed using the Release 10, 2007 of Stata Statistical Software and SPSS packages. Data will be subjected to a normality test (Shapiro-Wilk test). The means, standard deviations (SD) and medians (interquartile range) of the continuous data will be calculated. The frequency and percentage distributions of categorical data will be described. The reliability test (Cronbach alpha) will be done to measure the internal consistency of the questionnaires.

The nutrition knowledge scores of participants will be calculated based on a possible maximum of 60. Each subsection ‘s score of the nutrition knowledge questionnaire will be calculated and converted to percentages. The mean percentage of knowledge scores will also be calculated. Whati et al., performance rating scale of < 34% - very poor; (ii) 34 to 51% - fair/below average; (iii) 52 to 57% - good/average; (iv) 58 to 75% - very good/above average; and (v) 76 and above – excellent [45, 55] will be used to classify the nutrition knowledge performance.

Nutrition attitudes have eight dichotomous (agree, disagree) responses questions and the score will be the percentage of the total possible score (affirmative responses) using 60% as the cut-off for poor attitude performance [44].

The dietary practices will be classified based on how often participants consumed the FAO nine food groups [31] in the previous week. Participant’s lifestyle will also be classified based on the number of drinks containing alcohol and cigarettes consumption per day.

The construct of the QoL, nutrition knowledge and IDDS will be summarised by sex and age using percentages and 95% confidence intervals. Analysis of variance will be used to compare the intervention and control groups, to confirm the similarity in participant populations.

Multivariate regression analysis will be used for group comparison of overall QoL and the QoL constructs of the intervention and control groups. Other measurable outcomes will be compared (the baseline, week 12 and week 24) using baseline data as covariates. All the testing will be done at the 0.05 level of significance.

Atlas ti.8 software will be used for the qualitative data analysis. Focus group discussion will be recorded and transcribed verbatim by an independent person. To eliminate bias, the transcription will be coded by two independent researchers. Inconsistencies will be discussed until an agreement is reached.

## Discussion

This study protocol emerged from an international multidisciplinary collaborative study under the present South African Research Chair in Education and Care in Childhood which seeks to enhance the QoL of OVC by developing interventions programmes to reduce the factors of their vulnerability. It also aims to develop evidence-based education tools for increasing QoL, physical activity and general well-being of the OVC. The strengths of the study include the integration of qualitative and quantitative methods, objective and subjective measures, using previously validated tools [56–58]. This study seems to be the first to enhance protective factors among OVC with the support of their families/caregivers, schools and communities.

Other key strengths of this study include the involvement of researchers from diverse disciplinary backgrounds (education, public health, nutrition, and psychology researchers). Their inclusion will bring complementary perspectives to the challenges of OVC in place. The involvement of families/caregivers, schools and communities on the related outputs will contribute as much to the understanding of knowledge exchange.

The limitations of the study include the fact that a single province cannot be regarded as being representative of a nation or global section.

## Sustainability aspects of this study

- The involvement of stakeholders (the caregivers/families of the OVC) in the development of the NEP will enhance programme ownership and good will to support the continuation of the programme even after the study.
- Training the caregivers/families of the OVC using the educational materials and involving them in the delivery thereof will empower them to continue with the programme even after the study.

## Abbreviations

ENEP: Evidence-based nutrition education programme
FGD: Focus group discussion
HBM: Health belief model
KAP: Knowledge, attitude and practices
NEP: Nutrition education programme
OVC: Orphans and vulnerable children
QoL: Quality of life
SCT: Social cognitive theory
TFA: Thematic framework analysis
